# Nicotine Patch Alters Patterns of Cigarette Smoking-Induced Dopamine Release: Patterns Relate to Biomarkers Associated With Treatment Response

**DOI:** 10.1093/ntr/ntac026

**Published:** 2022-01-31

**Authors:** Yasmin Zakiniaeiz, Heather Liu, Hong Gao, Soheila Najafzadeh, Jim Ropchan, Nabeel Nabulsi, Yiyun Huang, David Matuskey, Ming-Kai Chen, Kelly P Cosgrove, Evan D Morris

**Affiliations:** Department of Psychiatry, Yale University, New Haven, CT, USA; Yale Positron Emission Tomography (PET) Center, Yale University, New Haven, CT, USA; Department of Biomedical Engineering, Yale University, New Haven, CT, USA; Yale Positron Emission Tomography (PET) Center, Yale University, New Haven, CT, USA; Department of Radiology and Biomedical Imaging, Yale University, New Haven, CT, USA; Yale Positron Emission Tomography (PET) Center, Yale University, New Haven, CT, USA; Department of Radiology and Biomedical Imaging, Yale University, New Haven, CT, USA; Yale Positron Emission Tomography (PET) Center, Yale University, New Haven, CT, USA; Department of Radiology and Biomedical Imaging, Yale University, New Haven, CT, USA; Yale Positron Emission Tomography (PET) Center, Yale University, New Haven, CT, USA; Department of Radiology and Biomedical Imaging, Yale University, New Haven, CT, USA; Yale Positron Emission Tomography (PET) Center, Yale University, New Haven, CT, USA; Department of Radiology and Biomedical Imaging, Yale University, New Haven, CT, USA; Department of Psychiatry, Yale University, New Haven, CT, USA; Yale Positron Emission Tomography (PET) Center, Yale University, New Haven, CT, USA; Department of Radiology and Biomedical Imaging, Yale University, New Haven, CT, USA; Yale Positron Emission Tomography (PET) Center, Yale University, New Haven, CT, USA; Department of Radiology and Biomedical Imaging, Yale University, New Haven, CT, USA; Department of Psychiatry, Yale University, New Haven, CT, USA; Yale Positron Emission Tomography (PET) Center, Yale University, New Haven, CT, USA; Department of Psychiatry, Yale University, New Haven, CT, USA; Yale Positron Emission Tomography (PET) Center, Yale University, New Haven, CT, USA; Department of Biomedical Engineering, Yale University, New Haven, CT, USA; Department of Radiology and Biomedical Imaging, Yale University, New Haven, CT, USA

## Abstract

**Introduction:**

Tobacco smoking is a major public health burden. The first-line pharmacological treatment for tobacco smoking is nicotine replacement therapy (eg, the nicotine patch (NIC)). Nicotine acts on nicotinic-acetylcholine receptors on dopamine terminals to release dopamine in the ventral and dorsal striatum encoding reward and habit formation, respectively.

**Aims and Methods:**

To better understand treatment efficacy, a naturalistic experimental design combined with a kinetic model designed to characterize smoking-induced dopamine release in vivo was used. Thirty-five tobacco smokers (16 female) wore a NIC (21 mg, daily) for 1-week and a placebo patch (PBO) for 1-week in a randomized, counter-balanced order. Following 1-week under NIC and then overnight abstinence, smokers participated in a 90-minute [^11^C]raclopride positron emission tomography scan and smoked a cigarette while in the scanner. Identical procedures were followed for the PBO scan. A time-varying kinetic model was used at the voxel level to model transient dopamine release peaking instantaneously at the start of the stimulus and decaying exponentially. Magnitude and spatial extent of dopamine release were estimated. Smokers were subcategorized by nicotine dependence level and nicotine metabolism rate.

**Results:**

Dopamine release magnitude was enhanced by NIC in ventral striatum and diminished by NIC in dorsal striatum. More-dependent smokers activated more voxels than the less-dependent smokers under both conditions. Under PBO, fast metabolizers activated more voxels in ventral striatum and fewer voxels in dorsal striatum compared to slow metabolizers.

**Conclusions:**

These findings demonstrate that the model captured a pattern of transient dopamine responses to cigarette smoking which may be different across smoker subgroup categorizations.

**Implications:**

This is the first study to show that NIC alters highly localized patterns of cigarette smoking-induced dopamine release and that levels of nicotine dependence and nicotine clearance rate contribute to these alterations. This current work included a homogeneous subject sample with regards to demographic and smoking variables, as well as a highly sensitive model capable of detecting significant acute dopamine transients. The findings of this study add support to the recent identification of biomarkers for predicting the effect of nicotine replacement therapies on dopamine function which could help refine clinical practice for smoking cessation.

## Introduction

Tobacco smoking is the world’s leading cause of preventable death. It is largely driven by the reinforcing effects of nicotine—the primary addictive chemical in cigarettes—which activates nicotinic acetylcholine receptors located on dopamine neurons to release dopamine in mesolimbic brain regions such as the striatum which encodes reward.^1,2^ Abstinence is hard to sustain. All available treatments for smoking cessation have limited success rates^3^ and most smokers relapse within 6 months of a quit attempt.^4^ One of the most widely-used smoking cessation treatments is the transdermal nicotine patch (NIC). NIC is a type of nicotine replacement therapy that releases a steady, low dose of nicotine that is absorbed through the skin. Just like cigarette smoking, NIC releases dopamine in reward regions of the brain. There is evidence that nicotine replacement therapies reduce cigarette use.^5^ It may be possible that NIC alters reward via its own effect on dopamine to reduce cigarette use, but smoking-induced dopamine release in the striatum during NIC treatment has not been thoroughly investigated. Certain traits, such as nicotine dependence level and nicotine clearance rate, have been identified as predictors of treatment efficacy. Higher nicotine consumption level is strongly associated with higher nicotine dependence and both are associated with poorer treatment outcomes.^6,7^ Nicotine metabolism ratio (NMR) has recently been identified as a biomarker for predicting the success of smoking cessation with NIC compared to varenicline, an FDA-approved smoking cessation treatment that modulates dopamine release.^8,9^

Several positron emission tomography (PET) studies have examined the dopamine response in vivo in tobacco smokers.^10–12^ These studies used amphetamine as a test stimulus that releases robust amounts of dopamine via several neurobiological mechanisms. Unlike amphetamine, nicotine or cigarettes release much smaller amounts of dopamine lasting only minutes.^13^ Some studies have investigated smoking-induced dopamine-release^14–18^ and a few other studies have assessed the smoking-induced dopamine response to cessation treatments.^19–21^ However, the results of these studies have been inconsistent. The inconsistencies may be attributed to: (1) an inability of conventional tracer kinetic models to detect small and short-lived dopamine responses, (2) a weakness of nicotine as a test-stimulus and/or, (3) an excessive delay between the stimulus and the PET scan. First, studies that use conventional time-invariant tracer kinetic models, such as simplified reference tissue model,^22^ measure the average smoking-induced changes in dopamine binding over the scan duration. The parametric endpoint used in these studies, *BP*_ND_ (receptor availability), is a steady-state parameter that fails to accurately capture the small and transient (on the order of minutes) alterations in the dopamine system elicited by cigarette smoking.^23^ Second, many routes of nicotine delivery including nicotine gum, NIC, IV nicotine, and cigarette smoking have been tried. Administering nicotine in unnatural ways (ie, other than cigarette smoking) may not produce a sufficiently robust dopamine response. Third, the dopamine response to cigarette smoking is brief so PET scanning too long after smoking may simply miss the response. In the present study, we used an appropriate model, a naturalistic stimulus, and a rigorous experimental design.

The current study is the first double-blind crossover design, including two PET scans per subject—one following 1-week on NIC and one following 1-week on a placebo patch (PBO)—to assess the effect of NIC on the striatal cigarette-induced dopamine response, at the voxel resolution. We evaluated whether nicotine dependence level (assessed as smoking pack-years), and nicotine clearance rate (assessed via NMR), affected NIC-induced changes to the transient dopamine response. We used motion-tracking technology and list-mode reconstruction to correct any intra-frame motion that might have occurred while subjects smoked inside the scanner during the scan.^24,25^ We also used the time-varying kinetic model LSRRM (linearized simplified reference region model),^26^ which models transient dopamine release peaking instantaneously at the start of the stimulus and decaying exponentially. Our two outcome measures were magnitude of dopamine release, and spatial extent of dopamine release in the precommissural striatum.^27^ We expected that the presence of NIC would alter the magnitude and location of the dopamine response to smoking, based on previous microdialysis and PET literature showing that repeated nicotine injections increase dopamine release in mesolimbic brain regions.^1,28,29^ We also expected that the putative biomarkers for treatment success (dependence level and nicotine metabolism rate) would affect the magnitude or spatial extent of the dopamine response, based on prior literature demonstrating the dopamine release was related to nicotine dependence^20^ and nicotine metabolism.^30,31^

## Materials and Methods

### Subjects

Thirty-five tobacco smokers (16 female) were studied. Subjects had no history or evidence of significant medical disorders on physical exam and did not meet Diagnostic and Statistical Manual of Mental Disorders, Fifth Edition (DSM-5) criteria for current or past psychiatric or substance abuse diagnosis (except nicotine dependence). Smokers on average (±SE) smoked 14 ± 1.4 cigarettes per day for 18 ± 2.3 years and had Fagerström Test for Cigarette Dependence (FTCD)^32,33^ scores of 5.1 ± 0.4, indicating moderate dependence (Table 1). On intake day, smoking status was confirmed by spirometry to measure carbon monoxide (CO) levels > 11 parts per million (ppm) and by urine samples to measure cotinine—the primary metabolite of nicotine—levels >150 ng/mL (NicAlert cotinine test strips; Nymox Pharmaceutical). On scan day, overnight abstinence was confirmed by CO levels < 10 ppm or ≤50% of their intake level. Pre-scan plasma nicotine and metabolites (cotinine and 3-hydroxy-cotinine) were collected. Pregnancy and lactation were exclusionary. Menstrual cycle phase was not controlled and use of hormonal contraception was not exclusionary.

**Table 1.** Group Demographics

	All subjects	Low PY	High PY	Low vs. High PY	Slow metabolizers	Fast metabolizers	Slow vs. fast metabolizers
N	35	17	18	—	15	16	—
	%	%	%	p	%	%	p
Sex (% male)	54	47	61	0.40	60	50	0.58
	Mean ± SE	Mean ± SE	Mean ± SE	p	Mean ± SE	Mean ± SE	p
Age (years)	36 ± 1.7	29 ± 1.6	43 ± 2.0	<<0.01*	35 ± 3.2	38 ± 2.2	0.59
Smoking measures							
Cigarettes/day	14 ± 1.4	11 ± 0.9	18 ± 2.3	0.02*	14 ± 2.1	15 ± 2.3	0.77
Years smoked	18 ± 1.6	11 ± 1.3	25 ± 1.6	<<0.01*	18 ± 2.6	19 ± 2.3	0.78
PY	14 ± 2.2	5.6 ± 0.6	22 ± 3.3	<<0.01*	14 ± 3.7	14 ± 3.3	0.90
FTCD	5.1 ± 0.4	3.9 ± 0.5	6.1 ± 0.6	0.01*	4.9 ± 0.6	5.2 ± 0.8	0.73
MNWQ	11 ± 1.6	8.6 ± 1.9	12 ± 2.6	0.26	9.3 ± 2.4	12 ± 2.6	0.50
QSU	35 ± 2.5	33 ± 3.4	36 ± 3.8	0.61	36 ± 3.8	34 ± 4.1	0.80
NMR	0.31 ± 0.02	0.30 ± 0.03	0.31 ± 0.03	0.40	0.20 ± 0.01	0.40 ± 0.02	<<0.01*

FTCD = Fagerström’s Test for Cigarette Dependence; MNWQ = Minnesota Nicotine Withdrawal Scale; NMR = nicotine metabolite ratio; PY = pack-years; QSU = Questionnaire of Smoking Urge; SE = standard error of the mean.

Subgroups were well-matched by sex and smoking measures. High pack-years subjects were older, smoked more cigarettes/day for more years, and scored higher on nicotine dependence than low pack-years subjects (*p* < .01). Fast metabolizers had higher NMR than slow metabolizers (*p* < .01). Pack-years and NMR groups were distinct, that is, pack-years groups did not differ in NMR and NMR groups did not differ in pack-years. Mean ± SE shown.

*p < .05.

### Study Design

The study was approved by the Yale Human Investigation and Radiation Safety Committees. Subjects wore a NIC (21 mg, daily) for 1-week and a PBO for 1-week in a double-blind, randomized, counterbalanced, placebo-controlled, cross-over design. Following 1-week on NIC and then overnight abstinence, smokers participated in a 90-minute [^11^C]raclopride PET scan while continuing to wear the NIC/PBO from the previous day. While lying in the scanner during continuous scanning they smoked a cigarette, as previously described.^24^ Briefly, subjects smoked one cigarette of their own brand, with their dominant hand, at their own pace (typically 3-min to complete a whole cigarette), starting at mid-session (35-min after scan start) without leaving the scanner. To remove secondhand smoke, an air filter (Movex, Inc., Northampton, PA) was positioned in front of the scanner and above the subject’s mouth for the entire scan duration. Identical procedures were followed for PBO condition. Smokers reported subjective ratings of craving, enjoyment of, and energized by the cigarette (on a 0–100 scale) pre- and post-cigarette smoking. Pre-scan withdrawal and craving measures were collected using the Minnesota Nicotine Withdrawal Scale (MNWQ)^34^ and Questionnaire of Smoking Urge (QSU),^35^ respectively.

### Subgroup Characterization

#### Pack-years

Smoking pack-years were calculated by multiplying the number of cigarette packs smoked per day by the number of years smoked. Participants were divided into low and high pack-years groups using a median split to assess the effects of nicotine dependence on smoking-induced dopamine release. Low and high pack-years smokers were matched for age, sex, MNWQ, and QSU scores (*p* > .05). High pack-years smokers were significantly older, smoked more cigarettes per day, smoked for more years, and had higher FTCD scores (*p* < .02) than the low pack-years smokers. We chose to use pack-years as a measure of dependence rather than FTCD for the following reasons: (1) FTCD has poor reliability and validity possibly due to dichotomous scoring,^32^ (2) FTCD does not account for the duration of smoking which is an important factor in the level of dependence, and (3) FTCD would not be appropriate for the current median split analysis because it is a categorical variable and thus, the resulting groups would not be as clinically meaningful.

#### NMR

NMR was calculated as the ratio between 3-hydroxy-cotinine and cotinine. Participants were divided into slow and fast NMR groups using the clinically-established cutoff NMR ratio of 0.31, based on^8^ to assess the effects of nicotine metabolism on smoking-induced dopamine release. Fast metabolizers had significantly higher NMR (*p* << .01) than slow metabolizers. Slow and fast metabolizers were matched for age, sex, and all other smoking characteristics (*p* > .2).

### Demographic Data Statistical Analysis

A chi-squared test was used to evaluate group differences in the categorical variable (sex). Student’s *t*-tests were used to evaluate group differences in continuous variables such as basic demographics (age), smoking questionnaires (eg, FTCD), and smoking measures (eg, cigarettes smoked per day) between conditions and between smoker subgroups.

### Imaging Data Acquisition

A 3T structural magnetic resonance imaging (MRI) scan for anatomical localization was collected from each subject (Trio and Prisma, Siemens Medical Systems, Erlangen, Germany). [^11^C]raclopride, a D_2/3_ antagonist, was synthesized as previously described in Ref. ^13^. Before each PET scan, a 6-min transmission scan was acquired for attenuation correction. [^11^C]raclopride was administered as a bolus by a computer-controlled pump (Harvard Apparatus, Holliston, MA) and collection of emission data for 90-min in 3-min time bins. The mean radioactivity dose was 19.2 ± 0.2 mCi (NIC: 19.5 ± 0.2 mCi; PBO: 19.0 ± 0.3 mCi; *p* = .34). PET was performed with the High-Resolution Research Tomograph (Siemens/CTI; FWHM = 2–3 mm).

Injected activity was compared between conditions and between smoker subgroups using student’s *t*-tests. To determine the effect of mass on the D_2_ receptor, we used the highest [^11^C]raclopride concentration (in mass units) in the reference region (cerebellum) and the affinity (k_D_) of raclopride for the D_2_ receptor,^36^ to calculate the largest possible occupancy of the D_2_ receptor by unlabeled raclopride (see Supplemental Material for the equation).

### Image Pre-processing and Post-processing

PET data were reconstructed using Motion-compensation OSEM (ordered subset expectation maximization) List-mode Algorithm for Resolution-recovery reconstruction (MOLAR),^37^ motion-corrected using Vicra (Polaris Vicra Tracking System; Northern Digital), and de-noised with a 3 × 3 × 3 voxel HighlY constrained backPRojection (HYPR) filter,^38,39^ according to the methods previously described.^24,39^ A 3D Gaussian filter (σ = 2 voxels) and was applied. PET data were aligned to the subject’s MRI and then spatially normalized to a standard MNI-152 template. A standardized 1004-voxel mask delineating the precommissural striatum and its subregions (left and right, ventral striatum [VS], dorsal caudate [DC], dorsal putamen [DP]), based on Martinez et al.,^27^ was applied to all subject images in template space prior to analysis.

The LSRRM model^26^ was fitted to the PET time-activity curve at each voxel. LSRRM parses the PET signal into a steady-state radiotracer component and a transient dopamine component. The transient component models dopamine release (starting and) peaking instantaneously at the start time of the stimulus (ie, cigarette smoking) and decaying exponentially.^40^

Significance^41^ was determined by model comparison between LSRRM and multilinear reference tissue model,^42^ using the Akaike information criterion corrected for small data sets.^43^ LSRRM estimates four parameters; multilinear reference tissue model (nested within LSRRM) estimates three. A cluster-size threshold (CST = 15 voxels) was applied to all significant voxels to correct for multiple comparisons.^24,41^ For a lengthy discussion of CST to correct for multiple comparisons, see paper by our group^41^ as well as others.^44–46^

### Creation of Parametric Images

Parametric images were created for two endpoints: magnitude of dopamine release and spatial extent of dopamine release.

1. *“Magnitude of Dopamine Release” Images.*Images of $\frac{\gamma }{k_{2a}}$ were produced, which represents a normalized magnitude of the transient dopamine response relative to the effective first-order efflux of the radiotracer. $k_{2a}$ is the efflux rate constant for efflux from the target tissue. It is in units of 1/time. γ is the magnitude of the peak transient dopamine release. But it also has units of inverse time. Thus, normalizing γ by $k_{2a}$($\frac{\gamma }{k_{2a}}$) yields a dimensionless parameter that can be compared across subjects. In effect, it represents the amount of additional efflux of tracer from the system that can be attributed solely to competition with transiently released dopamine.^47,48^ These images were produced for each subject and condition. The change in magnitude of release between conditions, $\delta \left(\frac{\gamma }{k_{2a}}\right),$ was calculated, defined as: $\;\;\delta (\frac{\gamma }{k_{2a}})=\frac{\gamma_{PBO}}{k_{2a_{PBO}}}-\frac{\gamma_{NIC}}{k_{2a_{NIC}}}$ where γ (magnitude of dopamine release) and $k_{2a}$ (radiotracer efflux rate constant from the target tissue) were estimated using LSRRM at each voxel. Binary maps of the voxels containing significant dopamine responses were created for each individual (see below). $\delta (\frac{\gamma }{k_{2a}})$ was compared between groups and conditions, by subregion.

Scans from 25 subjects were used to create “Magnitude of Dopamine Release” images. Three subjects were excluded because data were only available for one condition. Another subject was declared an outlier (>3 standard deviations from the mean number of activated voxels per subject on PBO) and removed. Six subjects did not have any voxels with a statistically significant level of detectable dopamine signal for either condition and their responses were deemed “below the level of detectability” (BLD) and excluded. All other subjects had at least one cluster with a significant dopamine response, for either condition.

2. *“Spatial Extent of Dopamine Release” Images.* Images were produced by summing the binary masks for all subjects and dividing by the number of subjects, for each condition. “Spatial Extent of Dopamine Release” induced by smoking was defined as the number of voxels activated by smoking and compared between groups and conditions, by subregion. All 35 subjects were used to create “Spatial Extent of Dopamine Release” images. “Spatial Extent of Dopamine Release” images were also created for low and high pack-years groups, and slow and fast metabolizer groups, for each condition. Statistical significance in the mean number of voxels activated by smoking in each subregion of the mask was assessed with a permutation test (two-tailed, *p* < .05, Bonferroni corrected by 1004 voxels in the mask), as previously described.^24^ For each permutation test, one hundred thousand random re-samplings scans were performed to achieve a zero mean difference between conditions in each subregion. Each set of re-samplings was used to create a null distribution for two arbitrary groups reflecting the actual sizes of the two experimental cohorts. To assess the likelihood of any given difference occurring by chance, we tested whether the mean value of the difference between PBO and NIC conditions was different from the zero (the null distribution) for each subregion. The null hypothesis of all permutations tests was that the mean difference in number of smoking-induced activated voxels between conditions was not different from zero, in a given subregion.

### Steady-State Parameters

Parametric images of steady state parameters, *R*_1_ and *BP*_ss_, are available from LSRRM. *R*_1_ describes the relative radiotracer delivery to the target region and *BP*_ss_ describes the receptor availability, absent the effects of the smoking stimulus. They were produced to compare the steady-state levels of radiotracer delivery and available dopamine receptors between conditions (see Supplemental Material for further explanation of the parameters and their images; Figures S2 and S3).

### Removal of the Order Effect

A significant order effect was detected in the magnitude of dopamine release whereby, [$mean\left(\frac{\gamma_{scan\;1}}{k_{2a\;scan\;1}}\right)>mean\left(\frac{\gamma_{scan\;2}}{k_{2a\;scan\;2}}\right)$, *p* < .05]. The order effect was removed. Figure S4 illustrates the process of removing the order effect from the images comparing NIC and PBO conditions (see Supplemental Material).

## Results

### Subjects

Twenty-eight (excluding outlier and BLD) smokers (13 female) were included in the final analysis. The exclusion of the outlier and BLD subjects did not change demographic or smoking measure data (*p* > .5), nor did it change any findings of significance between subgroups (Table S1).

### Smoking Characteristics

On average, subjects smoked less cigarettes per day during the week they were on NIC (10.9 ± 1, *p* = .002) and PBO (11.2 ± 1, *p* < .001) patches relative to baseline (at intake; 14.4 ± 1), demonstrating compliance with, and possibly effectiveness of, the patch protocol.

### Blood Measures

Plasma nicotine and cotinine levels were higher under NIC than PBO condition (*p* < .02), demonstrating compliance with the patch usage protocol (Table S2). NMR ratios were not different between conditions (*p* > .53). Smoking measures (CO levels, MNWQ, and QSU) were also not different between conditions (*p* > .41). The exclusion of outlier and BLD subjects did not impact significant and nonsignificant NIC versus PBO differences.

### Subjective Smoking Ratings

Craving scores decreased whereas enjoyment and energy scores increased following cigarette smoking, regardless of condition (Figure S1) or scan order (Table S3). Excluding outlier and BLD subjects did not impact average subjective ratings.

### Injection Parameters

On average, there were no significant differences in injected radiotracer activity between low and high pack-years, fast and slow metabolizers, first and second scans, or NIC and PBO scans (Table S4). The exclusion of BLD subjects did not impact significance of group differences in injected activity or mass. The largest calculated occupancy of the D_2_ receptor by unlabeled raclopride (mass) was only 5.7%.

### Validation of Steady-State Parameters

Steady-state parameter images were not different between conditions (see Figures S2 and S3 for images).

### Dopamine Metrics

####  “Magnitude of Dopamine Release” From Smoking by Condition

**Figure 1.**
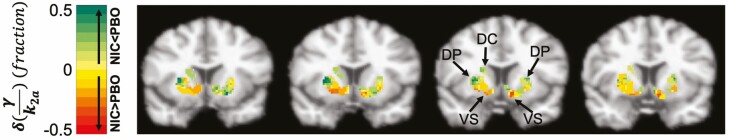
Change in magnitude of dopamine release (average$\;\;\delta (\frac{\gamma }{k_{2a}})$) images between PBO and NIC conditions across all subjects (*n* = 25). Dopamine release was increased by NIC compared to PBO in the VS. Dopamine release was decreased by NIC compared to PBO in the DC and DP. Regions are labeled in a representative slice. Right side of the brain is on the right. DC = dorsal caudate; DP = dorsal putamen; NIC = nicotine patch; PBO = placebo patch.

Figure 1 shows the average $\delta (\frac{\gamma }{k_{2a}})\;\;\;$ image. The average $\delta (\frac{\gamma }{k_{2a}})$, was negative in the VS, that is, dopamine release in bilateral VS was *increased* under the NIC compared to PBO condition (*p* < .05). The average $\delta (\frac{\gamma }{k_{2a}})$, was positive in DC and DP, that is, dopamine release in bilateral DC and bilateral DP was *decreased* under the NIC compared to PBO condition (*p* < .05). The overall pattern of $\delta (\frac{\gamma }{k_{2a}})$ was similar for male and female subjects.

####  “Spatial Extent of Dopamine Release” for Smoker Subgroups by Condition

**Figure 2.**
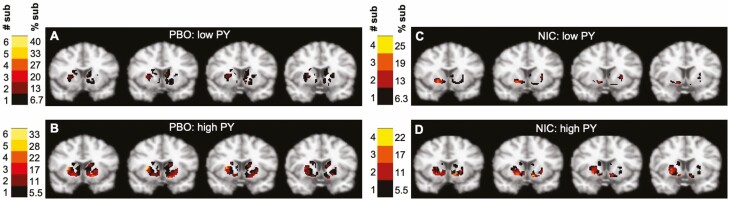
Spatial extent of dopamine release images for (A) low PY under PBO; (B) high PY under PBO; (C) low PY under NIC; (D) high PY under NIC. Color bar represents the number and percent of subjects (sub) with dopamine activation at each voxel. NIC = nicotine patch; PBO = placebo patch; PY = pack-years.

Figure 2 shows spatial extent of dopamine release images, split by condition and pack-years. The low pack-years group activated fewer voxels than the high pack-years group in both NIC and PBO conditions in the entire precommissural striatum, bilateral VS, bilateral DP, and left DC (*p* < .05). Within the low pack-years group, under PBO compared to NIC, fewer voxels were activated in bilateral VS (*p* < .05), whereas more voxels were activated in bilateral DC and left DP (*p* < .05). Within the high pack-years group, more voxels were activated under PBO compared to NIC in bilateral DC and bilateral DP (*p* < .05). See Figure S5 for mean number of activated voxels by condition and subregion for high and low pack-year groups.

**Figure 3.**
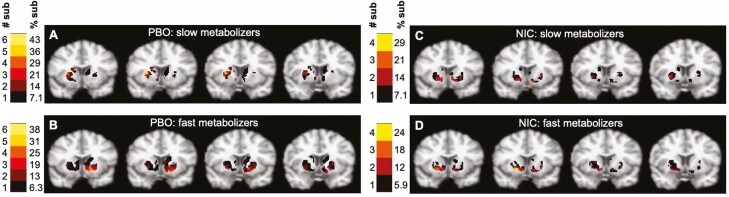
Spatial extent of dopamine release images for (A) slow metabolizers under PBO; (B) fast metabolizers under PBO; (C) slow metabolizers under NIC; (D) fast metabolizers under NIC. Color bar represents the number and percent of subjects (sub) with dopamine activation at each voxel. NIC = nicotine patch; PBO = placebo patch.

Figure 3 shows spatial extent of dopamine release images, split by condition and NMR. Slow metabolizers activated fewer voxels than the fast metabolizers under PBO, in the entire precommissural striatum (*p* < .05). Yet, the slow metabolizers activated more voxels than fast metabolizers under PBO in bilateral DP and left DC (*p* < .05). Among the slow metabolizers, more voxels were activated under PBO compared to NIC in the bilateral DC and bilateral DP (*p* < .05). Among the fast metabolizers, more voxels were activated under PBO compared to NIC in the bilateral VS, bilateral DC, and bilateral DP (*p* < .05). See Figure S6 for mean number of activated voxels by condition and subregion for fast and slow metabolizer groups.

## Discussion

### Compliance With Protocol and Summary of Findings

This is the first double-blind, randomized, placebo-controlled, cross-over study to examine cigarette-induced dopamine release following short-term nicotine replacement therapy. Smokers complied with the medication regimen, as evidenced by higher blood nicotine and cotinine levels under NIC condition compared to PBO and reduced smoking during the patch protocols relative to baseline, consistent with prior literature.^5^ In-scanner cigarette smoking decreased craving and increased enjoyment and energy ratings, as expected. Because subjective ratings of craving, enjoyment, and energy were not different between conditions, we did not predict that they would be related to brain changes. Subgroups and conditions were well-matched on injection parameters. This study has three main findings: (1) Among all smokers, the magnitude of dopamine release was enhanced by NIC in VS and diminished by NIC in DC and DP. (2) The more-dependent smokers activated more voxels than the less-dependent smokers under both conditions and less-dependent smokers activated more voxels in VS and fewer in DC and DP under NIC compared to PBO. (3) Fast and slow metabolizers activated more voxels under PBO compared to NIC in whole and dorsal striatum, respectively. Under the PBO condition only, fast metabolizers activated more voxels in VS and fewer voxels in DC and DP compared to slow metabolizers. Overall, our model captured mild, brief, and spatially localized dopamine responses to cigarette smoking which differed, regionally, by patch conditions and smoker subgroup categorizations, such as pack-years and nicotine metabolite ratio.

### Validation of Analysis Methods Using Steady-State Parameters

No change in steady-state parameters (*R*_1_ and *BP*_ss_) occurred between treatment conditions, throughout the striatum, as expected. *R*_1_ describes relative radiotracer delivery to the target region and *BP*_ss_ describes the receptor availability, absent the effects of the smoking stimulus. Both parameters represent the system at steady-state as opposed to effects of transient changes in available receptors caused by smoking-induced dopamine release. NIC may alter cigarette-induced dopamine release but is not expected to affect steady-state binding conditions appreciably. *R*_1_ and *BP*_ss_ images can be thought of as negative controls. These images (see Supplemental Material) increase our confidence that the spatially-varying pattern seen in the $\delta (\frac{\gamma }{k_{2a}})\;\;\;$ image (Figure 1) represents a true signal, rather than an artifact.

### Removal of the Scan Order Effect

We found a significant effect of scan order on the magnitude of dopamine release, such that greater cigarette-induced dopamine release was observed in the first scan session relative to the second, regardless of condition. This effect may be related to the novelty of the situation (ie, smoking a cigarette in the scanner) on the first scan.^49,50^ While this is an unintended effect of study design, the effect of order was successfully removed as evidenced by the nonsignificant difference in the magnitude of dopamine release between individuals with a NIC scan first relative to a PBO scan first (Figure S4).

### Spatially Varying Effect of NIC on Magnitude of Cigarette-Induced Dopamine Release

The ability to capture subtle fluctuations in endogenous dopamine concentration within a single-scan is novel and possible thanks to sophisticated modeling techniques. The presence of a time-varying term in LSRRM makes the model sensitive to brief dopamine competition with the radiotracer. Traditional kinetic models fail to capture transient disturbances of equilibrium caused by transient (eg, smoking-induced) dopamine release. Conventional time-invariant endpoints such as *BP*_ND_ assume a constant concentration of dopamine throughout the scan, and thus can only provide a crude estimate of changes in neurotransmitter level.^23^ An unintended consequence of using a time-invariant endpoint to describe a time-varying signal is that the endpoint is dependent on the duration of the scan.^7,23,51^ Relatively weak elevations in dopamine concentration, such as those caused by cigarette smoking, may be missed entirely. For example, studies have shown no dopamine release following a nicotine stimulus, for example, Ref.^52^, or no group differences in smoking-induced dopamine release, for example, Ref.^21^. The parameter presented herein, $\frac{\gamma }{k_{2a}},$ is analogous to *BP*_ND_ in the literature because it evaluates the change in the level of dopamine binding. However, $\frac{\gamma }{k_{2a}},$ is sensitive to short-lived changes in dopamine binding and is not dependent on scan duration. Thus, this endpoint has allowed us to capture and characterize nuances in the change in magnitude of the dopamine response caused by NIC, at voxel resolution.

We found that the magnitude of cigarette-induced dopamine release was greater under NIC compared to PBO condition in VS, suggesting an additive reinforcing effect of nicotine on the brain’s dopamine system. This is consistent with previous microdialysis and PET literature showing that a nicotine challenge following repeated daily injections increases dopamine release in a dose-dependent manner,^1,28,29^ regardless of the effects of treatment.^15^ Rollema et al. theorized that despite chronically elevated nicotine levels due to NIC, cigarette smoking still causes steep increases in nicotine levels and in dopamine release, thus, maintaining its reinforcing effect.^53^ This additive rewarding effect may be critical for driving smoking behavior and may decrease the likelihood of successful cessation. It is also important to note that subjects were overnight abstinent. Thus, the cigarette smoked in the scanner was the first cigarette of the day, which is the most pleasurable.^54^ Further, we found subregional variation in the effect of NIC on smoking-induced dopamine release consistent with previous reports. One study found that continuous microinjections of nicotine into the rat brain both increased and decreased [^11^C]raclopride dopamine release in the striatum, suggesting that local extracellular dopamine levels may have increased but extracellular levels of dopamine in the whole striatum may have decreased.^55^ An functional MRI study found that NIC increased smoking cue-induced activation in the caudate while decreasing putamen activation relative to PBO,^56^ supporting our findings. Heterogeneity in the spatial pattern of pharmacologically-induced dopamine release has also been reported for other drugs. For example, Yoder et al.^57^ found that individual dopamine responses to alcohol in the striatum were highly localized and varied widely by subject. Taken together, we can deduce that the pattern of cigarette-induced dopamine release is a complicated, highly localized phenomenon such that NIC treatment may not necessarily affect the striatum homogeneously, or even in a single direction.

### Differences in Spatial Pattern of Brain Response to Smoking by Dependence

Nicotine dependence level was related to cigarette-induced dopamine release. More-dependent smokers activated more voxels than less-dependent smokers regardless of the condition, suggesting that the longer people engage in their addictive behavior, the more of the striatum they must recruit to process rewarding and enjoyable stimuli. This is consistent with prior literature, for example, Ref. ^20^. Animal studies have demonstrated that repeated nicotine administration enhances psychomotor responses, the rewarding effects of nicotine, and striatal dopamine release in response to nicotine.^28^ In humans, nicotine gum-induced dopamine release in the VS was shown to be positively correlated with the degree of nicotine dependence.^20^

Our study also showed that more-dependent smokers activated more voxels in dorsolateral as opposed to ventral regions of the striatum than less-dependent smokers, suggesting a migration of dopamine activation from the goal-directed to the habit-formation striatum over time.^24,58^ We believe our finding links previous work in cocaine-dependent animals to nicotine-dependent humans who are dependent on nicotine for years as opposed to weeks/months in animals. Regardless of nicotine dependence level, smokers had a more widespread response across the dorsal striatum (albeit a lower magnitude) under PBO compared to NIC condition, suggesting that in response to a cigarette, more of the habit-related striatum was recruited when nicotine concentrations were lower in the blood, and presumably the brain.

### Difference in Spatial Extent of Brain Response by NMR

In the present work, nicotine clearance rate was associated with cigarette-induced dopamine release. Under PBO, fast metabolizers activated more voxels than slow metabolizers in the entire striatum, suggesting an enhanced dopamine response to cigarette smoking in individuals that clear nicotine faster. Fast metabolizers of nicotine experience a more rapid rate of absorption, entry of nicotine into the brain, a greater rush, and thus, greater reinforcement.^59^ This is consistent with studies showing that fast metabolizers have a greater dopamine or neural response than slow metabolizers when viewing smoking cues.^30,31^

One study previously examined cigarette-induced dopamine release between fast and slow metabolizers. No differences were found in post-cigarette [^11^C]-(+)-PHNO BP_ND_ between fast and slow metabolizers.^21^ However, this could be explained by a number of methodological differences between this study and our study including a less sensitive analysis method and a lengthy delay between cigarette smoking and PET scanning in the work by Di Ciano and colleagues. Fast metabolizers experience greater daily fluctuations in nicotine concentrations which may explain why they experience greater reward from smoking.^60^ Among slow metabolizers, NIC contributed to a greater spatial extent of dopamine response than PBO while the opposite was observed in the fast metabolizers in VS, DC, and DP. This finding could explain why, for slow metabolizers NIC is more efficacious whereas for fast metabolizers better smoking outcomes are achieved on therapies such as varenicline and bupropion, both of which increase dopamine concentration in the blood and presumably the brain.^8,59^ An improved understanding of the mechanisms underlying NMR associations with treatment response such as NIC, could help refine clinical practice for smoking cessation.

### Limitations

This study included a number of limitations that were primarily related to PET scanning and analyses.

#### Examination of Sex Differences

One of the goals of this study was to examine sex differences. We found that the overall pattern of $\delta (\frac{\gamma }{k_{2a}})\;\;\;$ was similar for male and female subjects when both NIC and PBO conditions are combined. However, due to small cell sizes, we were underpowered to examine sex differences in dopamine magnitude and spatial extent by individual NIC and PBO conditions.

#### Model Limitations

LSRRM models the dopamine response as starting (and peaking) at the time of stimulus and decaying exponentially thereafter. The assumption of an instantaneously-peaking response may be too restrictive for our data. Microdialysis experiments suggest that the peak magnitude of the dopamine response following a pharmacological stimulus could have a latency period, for example, Ref. ^53^. The true range of start-times and peak-times for the dopamine response is unknown and may vary based on treatment and subject. Our group has developed a suite of time-varying “ntPET” models that are specifically tailored to characterize the transient changes in neurotransmitter binding caused by a drug stimulus, for example, Ref. ^61^. We have used the lp-ntPET model, for example, Ref. ^41^ to not only detect, but also characterize brief smoking-induced dopamine transients at the voxel level—revealing novel sex differences in the spatiotemporal signature of the dopamine response.^24^ However, for the sake of parameter parsimony, we chose to model the dopamine response with a fixed start-time and peak-time with LSRRM. There is a tradeoff between sensitive detection and accurate characterization of the dopamine response. The reduced number of estimated parameters in LSRRM allows for greater detection sensitivity of the dopamine response, using the current significance testing approach that relies on model comparison.

#### Sparseness of Detected Dopamine Responses

This study was intended to capture the brief, small, dopamine response caused by a smoking stimulus. The transient nature of the dopamine response and sparseness of detected responses suggests that the smoking-induced dopamine response is at the limits of voxel-level detection with the current PET scanner technology. Due to the challenges in detection, each subject’s binary significance mask was sparsely populated within the precommissural striatum. That is, only a small fraction of subjects contained a significant $\frac{\gamma }{k_{2a}}$ at any given voxel. The sparseness of the aggregated dopamine release data limits the power of voxel-by-voxel (ie, image-level) analysis. We recognize that what we are presenting here are preliminary results of the effect of the NIC on the magnitude of dopamine response images in smokers who have a detectable dopamine response under NIC that is different from PBO. We note that the spatial extent maps included all subjects (even those without a detectable dopamine response) such that the probability of no activation under both conditions is accounted for. The lack of dopamine response in six individuals under both conditions suggests that these individuals may not benefit from nicotine replacement therapies such as NIC if, in fact, an appreciable dopamine response is important for treatment success.^62^

#### Statistical Significance of $\frac{\gamma }{k_{2a}}$ Image

No statistical differences in $\frac{\gamma }{k_{2a}}$ were found between subregions using the *t*-test or Wilcoxon sign-rank test (two-tailed). Figure 1 shows the average pattern of change in $\frac{\gamma }{k_{2a}}$ across subjects, however, subject-to-subject variability was high. Due to the sparseness of significant dopamine release at the voxel level, distributions of $\frac{\gamma }{k_{2a}}$ values contained heavy pile-up at zero at the voxel level, for both treatments and across subregions. Voxels within any subregion demonstrated a bimodal distribution of $\frac{\gamma }{k_{2a}}$ values, with the predominant peak at zero. Thus, subregional distributions of the change in $\frac{\gamma }{k_{2a}}$ overlap largely at zero.

## Conclusions and Implications

This is the first study to show that NIC alters highly localized patterns of cigarette smoking-induced dopamine release and that levels of nicotine dependence and nicotine clearance rate contribute to these alterations. This current work included a homogeneous subject sample with regards to demographic and smoking variables, an absence of comorbidities, and subject compliance with treatment, as well as a highly sensitive model capable of detecting significant acute dopamine transients. The findings of this study add support to recent identification of biomarkers for predicting the effect of nicotine replacement therapies on dopamine function.

## Supplementary Material

A Contributorship Form detailing each author’s specific involvement with this content, as well as any supplementary data, are available online at https://academic.oup.com/ntr.

ntac026_suppl_Supplementary_Material

ntac026_suppl_Supplementary_Data
