# Supplementary material for: Nicotine Patch Alters Patterns of Cigarette Smoking-Induced Dopamine Release: Patterns Relate to Biomarkers Associated With Treatment Response
Source: Nicotine Tob Res. 2022 Jan 31;24(10):1597–606. doi: 10.1093/ntr/ntac026 (PMC9575980; doi:10.1093/ntr/ntac026)
Supplement: ntac026_suppl_Supplementary_Material [file ntac026_suppl_supplementary_material.docx]

**Supplemental Material**

**1. Occupancy Formula**

**2. Subjective Ratings of Smoking** (Figure S1)

**3. Steady-state Parameters** (Figures S2 and S3)

**4. Order Effect Removal** (Figure S4)

**5. Group demographics** (Table S1)

**6. Condition Comparisons** (Table S2)

**7. Subjective Ratings of Smoking** (Table S3)

**8.** **Injection Parameters** (Table S4)

**9. Activated Voxels by Subgroup** (Figures S5 and S6)

**1. Occupancy Formula**

$Occupancy=\frac{[raclopride ]}{\left[ raclopride \right]+K_{D}}$ , where [raclopride] is the concentration of raclopride (nM) in the tissue and K_D_ is the equilibrium dissociation constant for raclopride at the D_2_ receptor (nM). We approximate the maximum occupancy by using the maximum molar concentration of raclopride in the cerebellum where the signal is assumed to be entirely free (and not bound) raclopride.

**2. Subjective Ratings of Smoking**


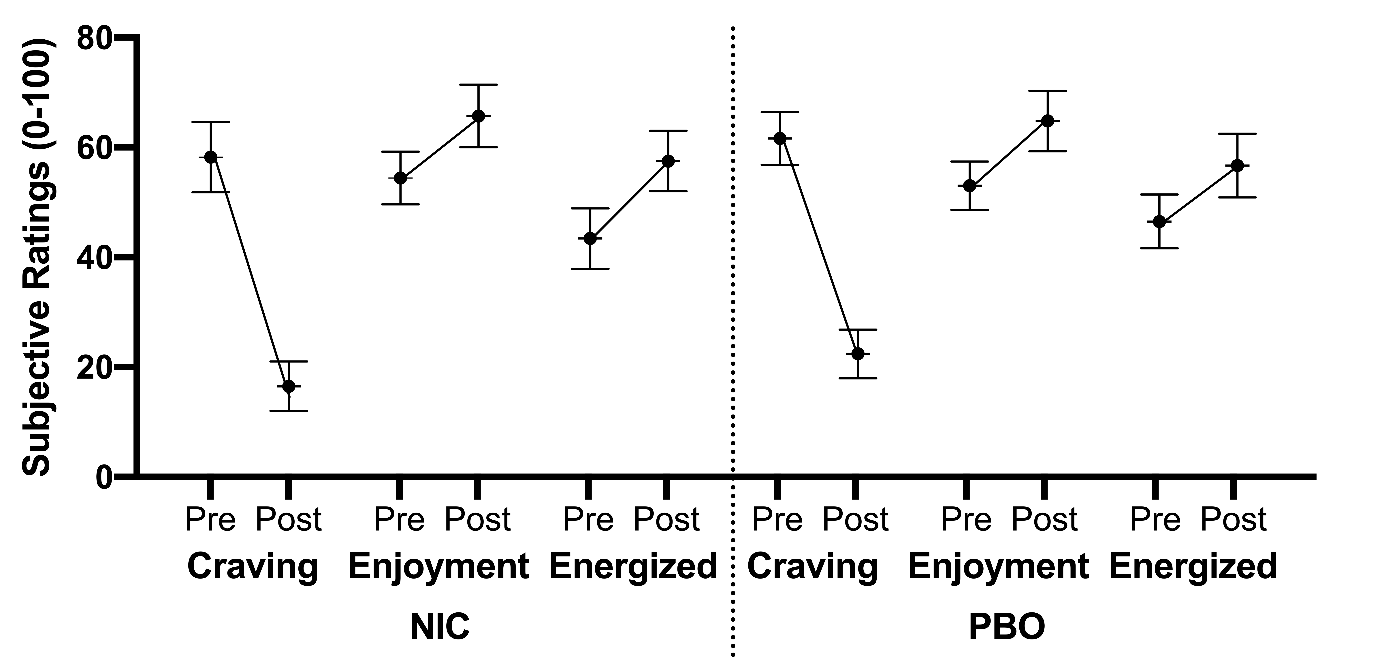


**Figure S1: Pre- and Post-smoking Subjective Ratings.** Smoking decreased craving and increased enjoyment and energy ratings under both NIC and PBO conditions (scale of 0-100) from pre-cigarette (1-min before cigarette smoking) to post-cigarette (3-min after the start of cigarette smoking). Abbreviations: NIC=nicotine patch, PBO=placebo patch.

**3. Steady-state parameters**

Parametric images showing the within-subject *change* between conditions in equilibrium parameters, *R_1_ (*ratio of radiotracer delivery to the target and reference tissues) and *BP_SS_* (level of specific binding by the tracer absent any stimulus) were produced. *BP_SS_* is defined as:$BP_{SS}=\frac{k_{2}}{k_{2a}}-1$ . To be clear, $BP_{SS}$ is a parameter of both NIC and PBO conditions, and does not describe any transient change in available receptors caused by dopamine release. It is thus a reflection of the steady-state level of available dopamine receptors.$\delta R_{1}$, is defined as $\delta R_{1}={R_{1}}_{PBO}-{R_{1}}_{NIC}$ and $\delta BP_{SS}$ is defined as $\delta BP_{SS}=B{P_{SS}}_{PBO}-{BP}_{SS_{NIC}}$, where, $R_{1}$ and $BP_{SS}$ are estimated from TACs at the voxel level from both the PBO and NIC scans. For consistency, the same cohort of 25 subjects used for creation of $\delta(\frac{\gamma}{k_{2a}})$ images was used for creation of $\delta R_{1}$ and $\delta BP_{SS}$ images. The average $\delta R_{1}$ and $\delta BP_{SS}$ images are shown in **Figures S2 and S3**, respectively.


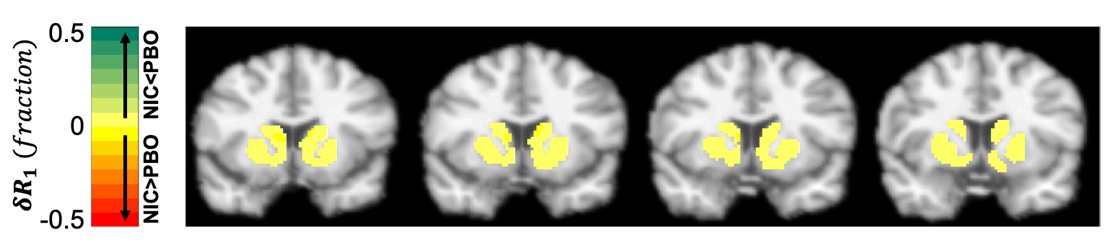


**Figure S2: Average** $\boldsymbol{\delta}\boldsymbol{R}_{\boldsymbol{1}}$ **images**. $\delta R_{1}$(fraction) between NIC and PBO conditions, averaged across subjects (n=25). Right side of brain is on right.


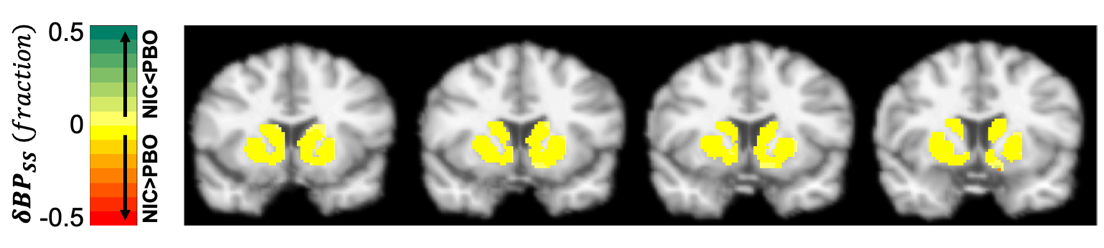


**Figure S3: Average** $\boldsymbol{\delta}\boldsymbol{B}\boldsymbol{P}_{\boldsymbol{SS}}$ **images.** $\delta BP_{SS}$(fraction) between NIC and PBO conditions, averaged across subjects (n=25).

**4. Order Effect Removal**

Removal of order effect (OE) in $\delta\left( \frac{\gamma}{k_{2a}} \right), \delta R_{1}$ and $\delta BP_{SS}$ images:

A two-tailed Wilcoxon signed-rank test was used to determine whether magnitude of the dopamine response differed between scan 1 and scan 2. The null hypothesis was $mean\left( \frac{\gamma_{scan 1}}{k_{2a scan 1}} \right)=$ $mean\left( \frac{\gamma_{scan 2}}{k_{2a scan 2}} \right)$, at each voxel. A significant OE was detected for [$mean\left( \frac{\gamma_{scan 1}}{k_{2a scan 1}} \right)>mean\left( \frac{\gamma_{scan 2}}{k_{2a scan 2}} \right)$, *p*<0.05].

The following procedure was applied to remove the OE from all images. **Step 1**: The strength of the OE (and its possible interaction with condition) was determined for subjects with a ‘PBO-first’ scan and subjects with a ‘NIC-first’ scan, separately. For PBO-first scans (n=13): the average of thirteen $\frac{\gamma}{k_{2a}}$ differences between a random Scan 1 and random Scan 2 pair was taken 50,000 times, at each voxel. For NIC-first scans (n=12): the average of twelve $\frac{\gamma}{k_{2a}}$ differences between a random Scan 1 and random Scan 2 pair was taken 50,000 times, at each voxel. **Step 2**: The mean condition-x-first images (created by taking the mean of the condition-x-first distribution at each voxel) were subtracted from the $\delta(\frac{\gamma}{k_{2a}})$ images created from the condition-x-first scans. The resultant two sets of images represent the $\delta(\frac{\gamma}{k_{2a}})$ signal with the OE removed. **Step 3:** To confirm that the OE was successfully removed from the $\delta(\frac{\gamma}{k_{2a}})$ signal from both PBO-first and NIC-first data, a two-tailed two-sample t-test was performed between $\delta(\frac{\gamma}{k_{2a}})$ values from the PBO-first and NIC-first images at each voxel, post-OE-subtraction. **Step 4**: Because there was no difference between the groups, PBO-first (n=13) and NIC-first (n=12) $\delta(\frac{\gamma}{k_{2a}})$ images were combined into a single data set of OE-corrected PBO – NIC images (n=25). Steps 1-4 were also applied to ${\delta R}_{1}$ and $\delta BP_{SS}$ images, for consistency.


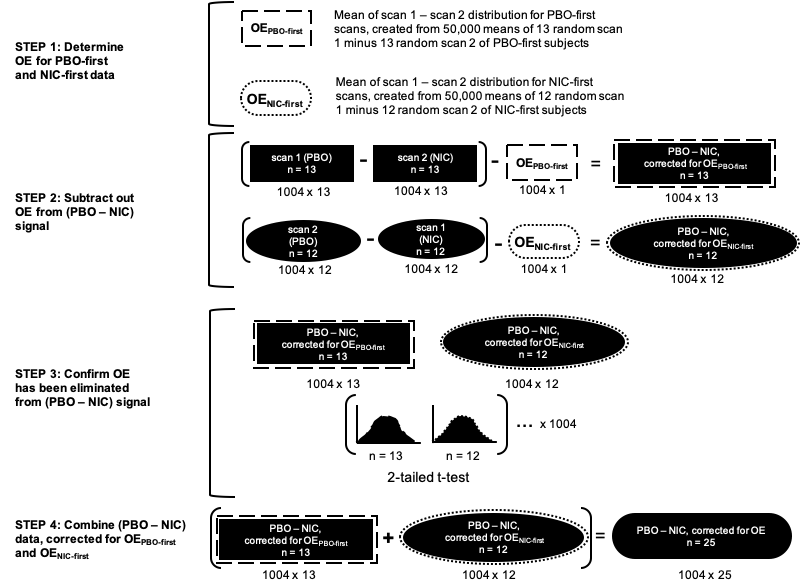


**Figure S4: Order Effect Removal.** Procedure for removing OE from parametric images. Each shape represents a matrix of image data, with dimensions specified beneath (first dimension = number of voxels; second dimension = number of images). The procedure was applied to $\delta(\frac{\gamma}{k_{2a}})$, $\delta R_{1}$, and $\delta BP_{SS}$ images.

**5. Group Demographics**

|  | **All subjects** | **All-BLD** | **All v. All-BLD** | **Low PY All**  **(All-BLD)** | **High PY All (All-BLD)** | **Low v. High PY All (All-BLD)** | **Slow Metab-olizers All (All-BLD)** | **Fast Metab-olizers All (All-BLD)** | **Slow v. Fast Metab-olizers All (All-BLD)** |
| --- | --- | --- | --- | --- | --- | --- | --- | --- | --- |
| **N** | **35** | **28** | - | **17 (13)** | **18 (15)** | - | **15 (13)** | **16 (13)** | - |
|  | **%** | **%** | ***P*** | **%** | **%** | ***P*** | **%** | **%** | ***P*** |
| Sex (% Male) | 54% | 54% | 0.96 | 47% (38%) | 61% (67%) | 0.40 (0.14) | 60% (53%) | 50% (62%) | 0.58 (0.69) |
|  | **Mean ± SE** | **Mean ± SE** | ***P*** | **Mean ± SE** | **Mean ± SE** | ***P*** | **Mean ± SE** | **Mean ± SE** | ***P*** |
| Age (years) | 36 ± 1.7 | 38 ± 2.1 | 0.59 | 29 ± 1.6  (29 ± 2.0) | 43 ± 2.0 (45 ± 2.0) | <<0.01* (<<0.01*) | 35 ± 3.2  (37 ± 3.5) | 38 ± 2.2  (38 ± 2.7) | 0.59  (0.77) |
| **Smoking Measures** | | | | | | | | | |
| Cigarettes/day | 14 ± 1.4 | 15 ± 1.7 | 0.77 | 11 ± 0.9  (11 ± 1.1) | 18 ± 2.3  (19 ± 2.7) | 0.02* (0.02*) | 14 ± 2.1  (14 ± 2.4) | 15 ± 2.3  (16 ± 2.7) | 0.77  (0.60) |
| Years Smoked | 18 ± 1.6 | 19 ± 1.8 | 0.68 | 11 ± 1.3  (11 ± 1.5) | 25 ± 1.6  (26 ± 1.6) | <<0.01* (<<0.01*) | 18 ± 2.6  (19 ± 2.8) | 19 ± 2.3  (19 ± 2.8) | 0.78  (0.94) |
| PY | 14 ± 2.2 | 15 ± 2.7 | 0.54 | 5.6 ± 0.6 (5.5 ± 0.7) | 22 ± 3.3  (24 ± 3.7) | <<0.01* (<<0.01*) | 14 ± 3.7  (15 ± 4.2) | 14 ± 3.3  (15 ± 4.0) | 0.90  (0.92) |
| FTND | 5.1 ± 0.4 | 5.4 ± 0.5 | 0.59 | 3.9 ± 0.5 (4.2 ± 0.6) | 6.1 ± 0.6 (6.4 ± 0.6) | 0.01* (0.02*) | 4.9 ± 0.6  (5.3 ± 0.5) | 5.2 ± 0.8  (5.5 ± 0.9) | 0.73  (0.85) |
| MNWQ | 11 ± 1.6 | 11 ± 2.0 | 0.77 | 8.6 ± 1.9 (7.5 ± 2.0) | 12 ± 2.6  (14 ± 2.8) | 0.26 (0.07) | 9.3 ± 2.4  (8.9 ± 2.7) | 12 ± 2.6  (14 ± 2.8) | 0.50  (0.20) |
| QSU | 35 ± 2.5 | 37 ± 2.9 | 0.62 | 33 ± 3.4  (35 ± 4.2) | 36 ± 3.8  (38 ± 4.1) | 0.61 (0.61) | 36 ± 3.8  (36 ± 4.3) | 34 ± 4.1  (38 ± 4.6) | 0.80  (0.80) |
| NMR | 0.31 ± 0.02 | 0.30 ± 0.02 | 0.84 | 0.30 ± 0.03 (0.30 ± 0.03) | 0.30 ± 0.03 (0.30 ± 0.03) | 0.40 (0.48) | 0.20 ± 0.01 (0.20 ± 0.02) | 0.40 ± 0.02 (0.40 ± 0.02) | <<0.01* (<<0.01*) |

**Table S1: Group demographics.** Excluding BLD subjects did not impact significant and non-significant subgroup differences on demographics and smoking measures. Mean±SE shown. Abbreviations:  All = all subjects, All-BLD = all subjects after excluding those "below detectable levels”, FTND = Fagerström’s Test for Nicotine Dependence, MNWQ = Minnesota Nicotine Withdrawal Scale, NMR = nicotine metabolite ratio,  PY = pack years, QSU = Questionnaire of Smoking Urge, SE = standard error of the mean, *p<0.05.

**6. Condition Comparisons**

|  | **NIC** | **PBO** | **NIC v. PBO** | |
| --- | --- | --- | --- | --- |
| **N** | **34** | **33** |  | |
|  | **Mean ± SE** | **Mean ± SE** | ***P*** | |
| **Smoking Measures** | | | |  |
| CO Level | 11 ± 1.5 | 12 ± 1.5 | **0.52** | |
| MNWQ | 12 ± 2.2 | 11 ± 1.8 | **0.41** | |
| QSU | 8.4 ± 0.9 | 8.4 ± 0.8 | **1.00** | |
| **Blood Measures** | | | |  |
| Nicotine (ng/ml) | 11 ± 1.4 | 4.1 ± 1.0 | **<<0.01*** | |
| Cotinine (ng/ml) | 381 ± 45 | 257 ± 27 | **0.01*** | |
| NMR | 0.32 ± 0.03 | 0.30 ± 0.03 | **0.53** | |

**Table S2: Condition Comparisons.** No significant differences in smoking measures were found between NIC and PBO conditions. Higher blood nicotine and cotinine levels were found under NIC vs. PBO condition (p≤0.01). Mean±SE shown. Abbreviations: CO = carbon monoxide, MNWQ = Minnesota Nicotine Withdrawal Scale, QSU = Questionnaire of Smoking Urge, NIC = nicotine patch, NMR = nicotine metabolite ratio, PBO = placebo patch, SE = standard error of the mean, *p<0.05.

**7. Subjective Ratings of Smoking**

|  | **All subjects** | **Scan 1** | **Scan 2** | | **Scan 1 v. Scan 2** | |  |
| --- | --- | --- | --- | --- | --- | --- | --- |
|  | **Mean ± SE** | **Mean ± SE** | **Mean ± SE** | | ***P*** | |  |
| **Subjective Ratings (Scale 0-100)** |  | | | |  | |  |
| Pre-Cigarette Craving NIC | 58 ± 6.4 | - | | - | | - | |
| Post-Cigarette Craving NIC | 16 ± 4.5 | - | | - | | - | |
| Pre-Cigarette Enjoy NIC | 54 ± 4.8 | - | | - | | - | |
| Post-Cigarette Enjoy NIC | 66 ± 5.7 | - | | - | | - | |
| Pre-Cigarette Energized NIC | 43 ± 5.5 | - | | - | | - | |
| Post-Cigarette Energized NIC | 58 ± 5.5 | - | | - | | - | |
| Pre-Cigarette Craving PBO | 62 ± 4.8 | - | | - | | - | |
| Post-Cigarette Craving PBO | 22 ± 4.4 | - | | - | | - | |
| Pre-Cigarette Enjoy PBO | 53 ± 4.4 | - | | - | | - | |
| Post-Cigarette Enjoy PBO | 65 ± 5.5 | - | | - | | - | |
| Pre-Cigarette Energized PBO | 47 ± 4.9 | - | | - | | - | |
| Post-Cigarette Energized PBO | 57 ± 5.8 | - | | - | | - | |
| Pre-Cigarette Craving NIC or PBO | - | 60 ± 5.7 | | 60 ± 5.6 | | **0.84** | |
| Post-Cigarette Craving NIC or PBO | - | 18 ± 4.1 | | 21 ± 4.8 | | **0.44** | |
| Pre-Cigarette Enjoy NIC or PBO | - | 55 ± 4.5 | | 53 ± 4.7 | | **0.44** | |
| Post-Cigarette Enjoy NIC or PBO | - | 62 ± 5.8 | | 69 ± 5.3 | | **0.34** | |
| Pre-Cigarette Energized NIC or PBO | - | 49 ± 4.8 | | 41 ± 5.5 | | **0.12** | |
| Post-Cigarette Energized NIC or PBO | - | 56 ± 5.7 | | 59 ± 5.6 | | **0.91** | |

**Table S3: Subjective Ratings of Smoking.** Mean values of subjective ratings of craving, enjoyment and energy. Rating means did not differ by scan order. Mean±SE shown. Abbreviations:  NIC = nicotine patch, PBO = placebo patch, PY = pack years, Metab = metabolizer, SE = standard error of the mean.

**8. Injection Parameters**

|  | **Low PY** | **High PY** | **Low v. High PY** | **Slow Metab-olizers** | **Fast Metab-olizers** | **Slow v. Fast Metab-olizers** | **Scan 1** | **Scan 2** | **Scan 1 v. Scan 2** | **NIC** | **PBO** | **NIC v. PBO** |
| --- | --- | --- | --- | --- | --- | --- | --- | --- | --- | --- | --- | --- |
|  | **Mean ± SE** | **Mean ± SE** | ***P*** | **Mean ± SE** | **Mean ± SE** | ***P*** | **Mean ± SE** | **Mean ± SE** | ***P*** | **Mean ± SE** | **Mean ± SE** | ***P*** |
| Injected Activity (MBq) | 716 ± 7.5 | 703 ± 9.4 | **0.35** | 715 ± 8.4 | 701 ± 13 | **0.39** | 700 ± 11 | 719 ± 9.1 | **0.28** | 722 ± 6.9 | 700 ± 12 | **0.13** |

**Table S4: Injection Parameters.** Injected activity was not different between subgroups, scan conditions, and scan order. Mean±SE shown. Abbreviations: NIC = nicotine, PBO = placebo, PY = pack years, SE = standard error of the mean.

**9. Activated Voxels by Subgroup** (Figures S5 and S6)

**
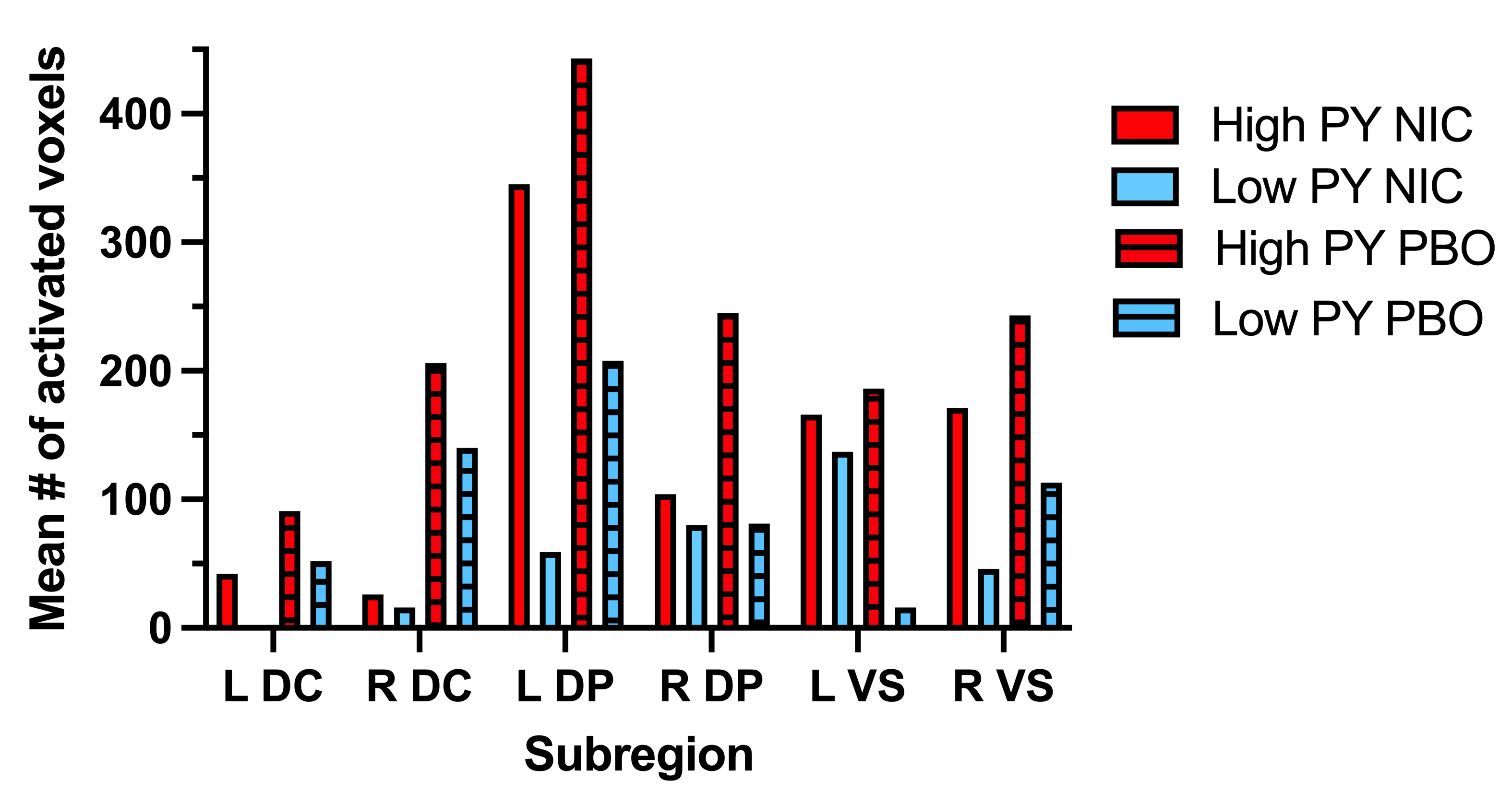
**

**Figure S5: Activated Voxels for High and Low Pack-Years Groups.** Mean number of activated voxels by subregion for high and low pack-years groups by NIC and PBO conditions. The low pack-years group activated fewer voxels than the high pack-years group in both NIC and PBO conditions in the entire precommissural striatum, bilateral VS, bilateral DP, and left DC (*p*<0.05). Within the low pack-years group, under PBO compared to NIC, fewer voxels were activated in bilateral VS (*p*<0.05), whereas more voxels were activated in bilateral DC and left DP (*p*<0.05). Within the high pack-years group, more voxels were activated under PBO compared to NIC in bilateral DC and bilateral DP (*p*<0.05).

**
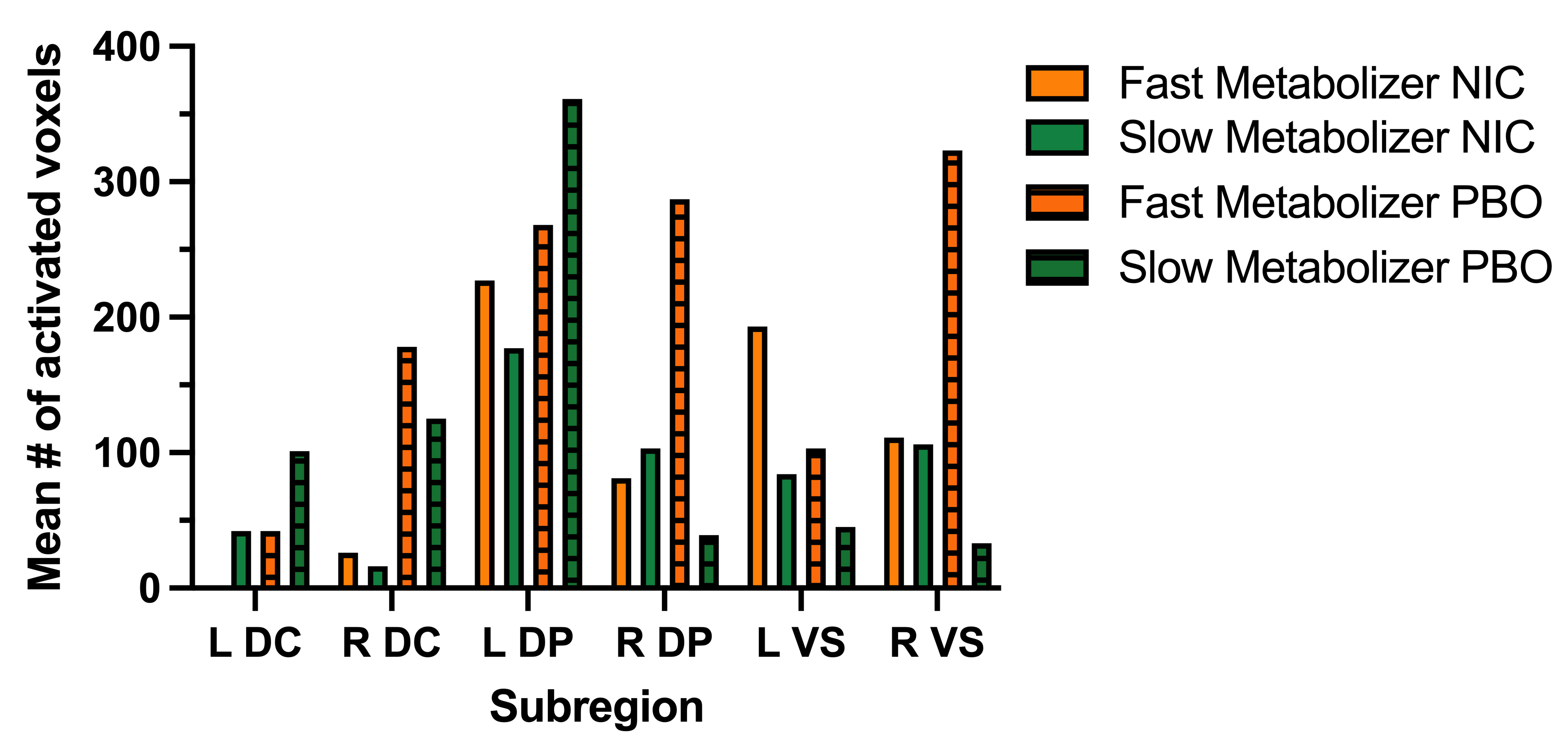
**

**Figure S6: Activated Voxels for Fast and Slow Metabolizer Groups.** Mean number of activated voxels by subregion for fast and slow metabolizer groups by NIC and PBO conditions. Slow metabolizers activated fewer voxels than the fast metabolizers under PBO, in the entire precommissural striatum (*p*<0.05). Yet, the slow metabolizers activated more voxels than fast metabolizers under PBO in bilateral DP and left DC (*p*<0.05). Among the slow metabolizers, more voxels were activated under PBO compared to NIC in the bilateral DC and bilateral DP (*p*<0.05). Among the fast metabolizers, more voxels were activated under PBO compared to NIC in the bilateral VS, bilateral DC, and bilateral DP (*p*<0.05).

Note: Error bars not shown for **Figures S5 and S6** because the variance derived from the resampling technique was used to determine statistical significance (see Materials and Methods, Section 2.7, Subsection (1)), not variance between the samples themselves. The sample does not meet assumptions of normality of data.
